# Adolescent Beliefs About Hookah and Hookah Tobacco Use and Implications for Preventing Use

**DOI:** 10.5888/pcd16.180093

**Published:** 2019-01-10

**Authors:** Molly Fitzpatrick, Andrea C. Johnson, Kenneth P. Tercyak, Kirsten B. Hawkins, Andrea C. Villanti, Darren Mays

**Affiliations:** 1 Lombardi Comprehensive Cancer Center, Georgetown University Medical Center, Washington, DC; 2 Milken Institute School of Public Health, George Washington University, Washington, DC; 3 Department of Pediatrics, MedStar Georgetown University Hospital, Washington, DC; 4 Vermont Center on Behavior and Health, Department of Psychiatry, University of Vermont Larner College of Medicine, Burlington, Vermont

## Abstract

**Introduction:**

Hookah tobacco use is popular among youths and there is evidence that perceived risks and normative beliefs are associated with hookah use. The aim of this study was to further examine associations between perceived risks of hookah use, normative beliefs, and lifetime hookah use among youths.

**Methods:**

Participants were adolescents aged 12 to 17 years (n = 257, mean [standard deviation] age, 14.9 [1.6] years, 40% nonwhite, 66% female) attending well-visit checkups at an urban pediatric clinic. Participants completed a survey of measures of cigarette smoking, risk factors for smoking, hookah use, perceived risks, and normative beliefs. Analyses examined associations among lifetime hookah use, beliefs about hookah use, and other smoking risk factors.

**Results:**

Overall, 15% of the sample had ever tried hookah smoking and 60% had ever tried cigarette smoking or were susceptible to cigarette smoking. Of those who had tried hookah smoking, 84% had also tried cigarettes or were susceptible to trying cigarettes (*P* < .001). One-third (33%) indicated that hookah smoking was less harmful than cigarettes, 38% indicated hookah smoking is less addictive than cigarettes, and 48% perceived that hookah smoking is somewhat or very socially acceptable among friends. In multivariable analyses adjusting for demographic and cigarette smoking–related factors, perceiving hookah use to be somewhat or very socially acceptable was associated with a significantly higher odds of ever having tried hookah smoking.

**Conclusion:**

The study findings indicate that stronger perceived social acceptability of hookah use is associated with a higher likelihood of trying hookah smoking among youths. These normative beliefs may be important targets of interventions aimed at preventing hookah use among youths.

**Summary**

**What is already known on this topic?**

Hookah tobacco use is popular among youths, and there is evidence that perceived risks and normative beliefs are associated with hookah use.

**What is added by this report?**

Greater perceived social acceptability of hookah tobacco use among friends was associated with higher odds of having ever tried hookah tobacco.

**What are the implications for public health practice?**

These normative beliefs may be important targets of interventions aimed at preventing youth hookah use. These findings can inform the development of interventions targeting the beliefs associated with youth hookah use as a prevention strategy.

## Introduction

Hookah tobacco use exposes users to high levels of harmful chemicals and is associated with short-term and long-term health effects, including cancer and lung disease (1). Nicotine exposure through hookah tobacco use produces dependence (2), and there is evidence that young people who try hookah smoking are more likely to initiate cigarette smoking (3). This suggests that factors that affect the risk of youth cigarette smoking (4) may also influence the risk of initiating hookah smoking.

Population data indicate that from 3.3% to 7.5% of US youths have tried hookah tobacco (5,6), but evidence of interventions to prevent hookah use initiation is limited (7). In the context of comprehensive tobacco control efforts, interventions such as mass media campaigns and education messaging targeted toward youths could be expanded to address youth hookah tobacco use (8).

Research examining factors associated with youth hookah use is critical to guide intervention development. Behavioral beliefs, including perceived risks and normative beliefs, are potentially modifiable preventive intervention targets. Some studies indicate that youths view hookah smoking to be equally or more harmful and addictive than cigarettes, while others demonstrate that youths view hookah smoking as less harmful and addictive than cigarettes (9). Normative beliefs are consistently associated with youth hookah use (9), but there is limited research on the independent associations of perceived risks and normative beliefs with youth hookah use behavior when examined concurrently and while accounting for other factors that may influence the risk of using hookah tobacco (4).

This study examined associations between perceived risks of hookah use, normative beliefs, and lifetime hookah use in a sample of youths aged 12 to 17 years. We hypothesized that after accounting for factors associated with cigarette smoking, youths who perceive hookah tobacco to be less risky than cigarettes and more socially acceptable will be more likely to have ever tried hookah tobacco.

## Methods

### Sample and procedures

This study analyzed data that were collected as part of a larger study of cigarette smoking prevention messaging among youths (N = 319) conducted in 2013 through 2015 in Washington, DC (7). Briefly, participants were offered the option of visiting a tobacco use prevention website after completion of the baseline survey; a follow-up survey was conducted 1 month later. Data on hookah tobacco use, hookah tobacco beliefs, and other factors were added as part of a study follow-up assessment and collected for 81% of the original study sample.

Adolescents aged 12 to 17 years (n = 257) attending well-visit checkups at an urban pediatric clinic were recruited to participate. Eligible participants were within the study age range, had internet access, and had access to an email address to complete the study procedures. All participants provided signed assent and parental consent. Study procedures were reviewed and approved by the Georgetown University Institutional Review Board.

Enrolled participants completed a confidential online assessment. The baseline assessment included measures assessing demographic characteristics, cigarette smoking, and risk factors for cigarette smoking. The follow-up assessment 1 month after baseline asked about hookah use and beliefs about hookah tobacco.

### Measures

Demographic characteristics assessed were participants’ sex, race, ethnicity, and age. We captured data on adolescents’ risk of cigarette smoking by using 2 variables: lifetime cigarette smoking and cigarette smoking susceptibility. Lifetime cigarette smoking was measured by using a valid item from adolescent tobacco surveys (10), and adolescents were categorized into never smokers and those who had tried cigarette smoking (11–13). Data on cigarette smoking susceptibility among never smokers were captured by using a valid 4-item measure assessing the likelihood that youths will try cigarette smoking under various scenarios in the future (11). This measure identifies nonsmokers who are at risk of smoking initiation and is predictive of youth cigarette smoking behavior (11). By using these measures, we created a binary variable indicating if participants 1) were susceptible to cigarette smoking in the future or had ever tried cigarette smoking or 2) were not susceptible and had never tried cigarette smoking (7). Susceptible never smokers and ever smokers were combined because of the low prevalence of ever smoking in the sample (11.7%).

Established risk factors for adolescent cigarette smoking were measured to account for their potential influence on hookah use behavior (4). Exposure to others’ cigarette smoking was measured with items assessing cigarette smoking by parents and among male and female friends (14,15). These were used to create 2 variables indicating if any parents or friends smoked cigarettes (yes/no). Frequency of exposure to tobacco advertising in movies, the internet, print media, and point of sale was measured with 4 items adapted from a previously validated adolescent tobacco use survey (16,17). Items were summed to create a score, with higher values indicating more frequent tobacco advertising exposure (range, 4–20).

Perceived harm and addictiveness of hookah smoking compared with cigarettes were measured by using a single item each. Response options were much less, less, about the same, more, or much more harmful or addictive (18). For analyses, responses were grouped into dummy variables indicating perceptions that hookah smoking is as about the same, more harmful or addictive than cigarettes, or less harmful or addictive than cigarettes.

Perceived peer use and social acceptability of hookah use were measured with a single item each (19). Perceived peer hookah use was assessed by asking how many peers at school have ever smoked hookah with the following response options: none, very few, about half, more than half, most, or all. Data on social acceptability were captured by asking how acceptable participants thought it was to smoke hookah tobacco among their friends, with response options not acceptable, somewhat acceptable, or very acceptable. For analyses, the items for perceived peer hookah use were grouped into dummy variables none or very few, about half, more than half, most, or all. Social acceptability was analyzed based on the response categories for not acceptable, somewhat acceptable, or very acceptable.

Lifetime hookah use was assessed by using a valid item (10) asking whether participants had ever tried hookah smoking, even 1 or 2 puffs (yes/no). We measured lifetime use as an indicator of hookah tobacco initiation among youths to identify potential targets for primary prevention.

### Statistical analysis

Sample characteristics were examined by using descriptive statistics. Bivariate analyses were used to assess associations between variables measured and the dependent variable of lifetime hookah use. A multivariable logistic regression model was then created where characteristics associated with lifetime hookah use in the bivariate analyses at *P* less than .05 were included as independent variables to examine multivariable associations with ever hookah use (yes/no) as the dependent variable. The Hosmer-Lemeshow test was used to assess goodness-of-fit for the model. All analyses used SAS version 9.4 (SAS Institute, Inc).

## Results

Overall, 15% had ever tried hookah tobacco and 60% had either tried cigarette smoking or were susceptible to cigarette smoking (Table 1). One-third of participants viewed hookah smoking to be less harmful than cigarettes, and 38% viewed it to be less addictive than cigarettes. Nearly 40% of participants reported that half or more of their peers use a hookah, and 48% perceived hookah smoking to be somewhat or very socially acceptable (Table 1).

**Table 1:** Characteristics of Adolescents (n = 257) Aged 12 to 17 Years Asked About Hookah and Hookah Tobacco Use, Washington, DC, 2013–2015^a^

Characteristic	Value^b^
**Sex**	
Male	88 (34.2)
Female	169 (65.8)
**Race**	
White	154 (59.9)
Black	55 (21.4)
Other race	48 (18.7)
**Ethnicity**	
Hispanic	33 (12.8)
Non-Hispanic	224 (87.2)
**Age, mean (SD), y**	14.9 (1.6)
**Adolescent cigarette smoking risk**	
Tried smoking or susceptible never smoker	153 (60.0)
Not susceptible, never smoker	102 (40.0)
**Parents smoke cigarettes**	
Yes	26 (10.1)
No	231 (89.9)
**Friends smoke cigarettes**	
Yes	71 (27.6)
No	186 (72.4)
**Tobacco advertising exposure, mean (SD)^c^**	11.6 (3.0)
**Perceived harms of hookah**	
Less harmful than cigarettes	84 (33.1)
About the same as cigarettes	110 (43.3)
More harmful than cigarettes	60 (23.6)
**Perceived addictiveness of hookah smoking**	
Less addictive than cigarettes	97 (38.0)
About the same as cigarettes	123 (48.2)
More addictive than cigarettes	35 (13.7)
**Perceived peer hookah use**	
More than half, most, or all	45 (17.7)
About half	53 (20.8)
None or very few	157 (61.6)
**Social acceptability of hookah use**	
Very acceptable	38 (15.0)
Somewhat acceptable	83 (32.7)
Not acceptable	133 (52.4)
**Ever tried hookah**	
Yes	38 (14.8)
No	219 (85.2)

Abbreviation: SD, standard deviation.

a Data displayed are n (%) unless otherwise indicated.

b Some totals do not sum to total sample n because of sporadic missing data (<5% for any given variable).

c Range, 4–20.

Those who had tried hookah smoking were on average older, most had tried cigarette smoking or were susceptible to trying cigarette smoking, and most had friends who smoked cigarettes (Table 2). Those who had ever tried hookah smoking perceived it to be less addictive than cigarettes, perceived greater peer hookah use, and perceived hookah use to be more socially acceptable than those who had never tried hookah smoking (Table 2).

**Table 2:** Bivariate Associations With Ever Trying Hookah Tobacco Among Adolescents (n = 257) Aged 12 to 17 Years Asked About Hookah and Hookah Tobacco Use, Washington, DC, 2013–2015

Characteristic	Ever Tried Hookah Smoking^a^		*P* Value
	Yes	No	
**Sex**			
Male	13 (34.2)	75 (34.2)	>.99
Female	25 (65.8)	144 (65.8)	
**Race**			
White	26 (68.4)	128 (58.5)	.18
Black	9 (23.7)	46 (21.0)	
Other race	3 (7.9)	45 (20.5)	
**Ethnicity**			
Hispanic	5 (13.2)	28 (12.8)	.95
Non-Hispanic	33 (86.8)	191 (87.2)	
**Age, mean (SD), y**	16.2 (0.87)	14.7 (1.6)	< .001
**Adolescent cigarette smoking risk**			
Tried smoking or susceptible never smoker	32 (84.2)	121 (55.8)	.001
Not susceptible, never smoker	6 (15.8)	96 (44.2)	
**Parents smoke cigarettes**			
Yes	8 (21.0)	18 (8.2)	.02
No	30 (79.0)	201 (91.8)	
**Friends smoke cigarettes**			
Yes	24 (63.2)	47 (21.5)	< .001
No	14 (36.8)	172 (78.5)	
**Tobacco Advertising Exposure, mean (SD)^b^**	12.4 (2.6)	11.5 (3.1)	.10
**Perceived harms of hookah**			
Less harmful than cigarettes	11 (29.7)	73 (33.6)	.08
About the same as	12 (32.4)	98 (45.2)	
More harmful than cigarettes	14 (37.8)	46 (21.2)	
**Perceived addictiveness of hookah smoking**			
Less addictive than cigarettes	23 (60.5)	74 (34.1)	.008
About the same as	12 (31.6)	111 (51.2)	
More addictive than cigarettes	3 (7.9)	32 (14.8)	
**Perceived peer hookah use**			
More than half, most, or all	16 (42.1)	29 (13.4)	<.001
About half	9 (23.7)	44 (20.3)	
None or very few	13 (34.2)	144 (66.4)	
**Social acceptability of hookah use**			
Very acceptable	18 (47.4)	20 (9.3)	<.001
Somewhat acceptable	17 (44.7)	66 (30.6)	
Not acceptable	3 (7.9)	130 (60.2)	

Abbreviation: SD, standard deviation.

a Data displayed are n (%) unless otherwise indicated. Some totals do not sum to total sample n because of sporadic missing data (<5% for any given variable).

b Range, 4–20.

The multivariable logistic regression model including factors associated with ever trying hookah smoking at *P* less than .05 in bivariate analyses fit the data well (Hosmer-Lemeshow [8 *df*] = 6.37, *P* = .61) (Table 3). Controlling for other variables in the model, the odds of having ever tried hookah tobacco increased with age (adjusted odds ratio [aOR], 1.60; 95% confidence interval [CI], 1.09–2.35), were greater among those who had ever tried cigarette smoking or were susceptible to trying cigarette smoking (aOR, 2.97; 95% CI, 1.03–8.56), and were greater among those who reported having parents who smoke cigarettes (aOR, 5.41; 95% CI, 1.54–19.02). Controlling for other variables in the model, we found an increase in the odds of having ever tried hookah tobacco among those who viewed hookah tobacco use as somewhat (aOR, 5.70; 95% CI, 1.37–23.77) or very socially acceptable (aOR, 12.36; 95% CI, 2.61–58.50), compared with those who perceived hookah tobacco use as not socially acceptable.

**Table 3:** Logistic Regression Analysis of Correlates of Ever Trying Hookah Tobacco Among Adolescents (n = 257) Aged 12 to 17 Years Asked About Hookah and Hookah Tobacco Use, Washington, DC, 2013–2015

Characteristic	Adjusted Odds Ratio (95% Confidence Interval)	*P* Value
**Age**	1.60 (1.09–2.35)	.02
**Adolescent cigarette smoking risk**		
Tried smoking or susceptible never smoker	2.97 (1.03–8.56)	.04
Not susceptible, never smoker	1 [Reference]	
**Parents smoke cigarettes**		
Yes	5.41 (1.54–19.02)	.009
No	1 [Reference]	
**Friends smoke cigarettes**		
Yes	1.68 (0.68–4.17)	.27
No	1 [Reference]	
**Perceived addictiveness of hookah smoking**		
Less addictive than cigarettes	2.90 (0.55–15.40)	.21
About the same as	1.32 (0.25–7.05)	.74
More addictive than cigarettes	1 [Reference]	
**Perceived peer hookah use**		
More than half, most, or all	1.68 (0.57–5.00)	.35
About half	0.84 (0.29–2.44)	.75
None or very few	1 [Reference]	
**Social acceptability of hookah use**		
Very acceptable	12.36 (2.61–58.50)	.002
Somewhat acceptable	5.70 (1.37–23.77)	.02
Not acceptable	1 [Reference]	

## Discussion

This study examined associations among perceived risks of hookah tobacco use, normative beliefs, and lifetime hookah tobacco use in a convenience sample of youths aged 12 to 17 years. The findings indicate that, in multivariable models accounting for other demographic characteristics and cigarette smoking–related factors, greater perceived social acceptability of hookah tobacco use among friends was associated with higher odds of having ever tried hookah tobacco. Lower perceived addictiveness of hookah tobacco compared with cigarettes was associated with having ever tried hookah smoking in bivariate analyses but not in the multivariable analysis. Perceived harms of hookah smoking compared with smoking cigarettes were not associated with having ever tried hookah smoking in bivariate or multivariable analyses. The findings of this study can help to inform the development of interventions aimed at preventing youth hookah tobacco use.

This study adds to the evidence on factors associated with youth hookah tobacco use in several ways. Prior research on beliefs among youths about the harms and addictiveness of hookah tobacco use is somewhat mixed (9). Although some studies demonstrate that youths perceive hookah tobacco to be comparable to cigarette smoking in terms of health harms, others report divergent results (9). Many studies demonstrate that youths tend to hold beliefs that hookah tobacco use is not addictive and they can quit at any time (9). In our sample, although most youths viewed hookah tobacco as equally or more harmful and addictive than cigarettes, one-third or more endorsed views that hookah tobacco is less harmful and less addictive than cigarettes. However, these beliefs about risks were not significantly associated with lifetime hookah use in multivariable analyses. Studies with young adults demonstrate that public health messages communicating the harms and addictiveness of hookah tobacco may be effective to prevent use initiation among young adult nonusers (20). Our findings indicate that messages targeting youths that are limited to communicating potential harms and addictiveness of hookah tobacco alone may have limited effectiveness for prevention. Considering other factors such as perceived social acceptability may help to improve the effectiveness of messages for prevention among youths.

Messaging campaigns to prevent tobacco use among youths that have improved behavioral outcomes integrate messages targeting multiple themes that affect tobacco use behavior among youths (21). The key finding of our study is that as participants’ perceived social acceptability of hookah tobacco use among their friends increased, the odds of having ever tried hookah tobacco increased by more than 5-fold. Hookah tobacco is often used in social settings with peers (22), and research conducted in diverse geographical settings demonstrates the influence of peers on youth hookah use (20). This finding is also consistent with studies among young adults, highlighting the role of normative beliefs and peer influence on hookah initiation and use (9). In addition to other known risk factors (23), social acceptability was found in our study to be a content area to examine in future studies seeking to develop interventions aimed at preventing youth hookah use. The outcome we examined was whether youths had ever tried hookah tobacco, suggesting such messages may be optimally targeted toward nonuser populations as a strategy for primary prevention. This targeting is an important avenue for future research given the limited available evidence on interventions to prevent youth hookah use (18).

The tobacco regulatory context in the United States creates opportunities to address youth hookah use through such interventions. In 2016, the Food and Drug Administration (FDA) finalized the regulations expanding FDA’s tobacco regulatory authority to include hookah and other tobacco products (24). The 2016 regulations subject hookah tobacco to many of the regulations of the Family Smoking Prevention and Tobacco Control Act (eg, minimum age of sale, prohibitions on youth-oriented marketing and promotions), and position FDA to engage in public education messaging alongside other public health agencies to communicate the risks of hookah use to youths. The Centers for Disease Control and Prevention also seeks to educate the public about the risks of hookah tobacco through online communications (25,26). These materials could be optimized or delivered in various contexts with these results in mind as well.

Research is needed to identify the optimal channels for delivering and engaging youths with hookah tobacco prevention messaging. Tobacco prevention media campaigns targeting youths have leveraged multiple media, including social media (21,27). Messages delivered online that communicate the health risks associated with cigarette smoking can motivate youths to engage with online smoking prevention content (7); however, our findings indicate that messages that target youth hookah use would be best positioned by integrating content targeting their beliefs about social acceptability and potentially other constructs. Evidence suggests social media where youths spend time and engage with content is a prominent source of messages promoting hookah tobacco (28,29). Messages promoting hookah tobacco through social media often include themes normalizing social aspects of hookah tobacco use and promote features that appeal to youths, such as flavoring, yet social media channels infrequently include messaging on hookah use prevention (28,29). In addition to investigating message content, examining message delivery channels that appeal to and engage youths, such as social media for the delivery of hookah tobacco use prevention messaging, is an important avenue for future research.

Our findings should be interpreted in light of limitations of this study. The study included a convenience sample of youths recruited from a single geographic location, limiting generalizability of the findings to other populations. In the parent study from which our data were drawn, participants lost to follow-up were mostly black, had parents who smoked, and had greater exposure to tobacco advertising at baseline than others in the study (7), which may affect our findings. Study participants also had an opportunity to visit a tobacco use prevention website, which may have affected their responses. The cross-sectional data do not allow for inferences about causal associations among hookah tobacco beliefs and the behavior examined. All measures were based on participant self-report. Although valid measures were used, they are subject to potential reporting biases.

Despite these limitations, our findings indicate that perceived social acceptability of hookah tobacco use is associated with lifetime use of hookah among youths when taking into account demographic, cigarette smoking, and other hookah-related covariates. These findings can inform the development of interventions targeting the beliefs associated with youth hookah use as a prevention strategy.

## References

[1] Cobb C , Ward KD , Maziak W , Shihadeh AL , Eissenberg T . Waterpipe tobacco smoking: an emerging health crisis in the United States. Am J Health Behav 2010;34(3):275–85. 10.5993/AJHB.34.3.3 20001185 PMC3215592

[2] Aboaziza E , Eissenberg T . Waterpipe tobacco smoking: what is the evidence that it supports nicotine/tobacco dependence? Tob Control 2015;24(Suppl 1):i44–53. 10.1136/tobaccocontrol-2014-051910 25492935 PMC4345797

[3] Watkins SL , Glantz SA , Chaffee BW . Association of noncigarette tobacco product use with future cigarette smoking among youth in the Population Assessment of Tobacco and Health (PATH) study, 2013–2015. JAMA Pediatr 2018;172(2):181–7. 10.1001/jamapediatrics.2017.4173 29297010 PMC5801043

[4] Wellman RJ , Dugas EN , Dutczak H , O’Loughlin EK , Datta GD , Lauzon B , Predictors of the onset of cigarette smoking: a systematic review of longitudinal population-based studies in youth. Am J Prev Med 2016;51(5):767–78. 10.1016/j.amepre.2016.04.003 27180028

[5] Kasza KA , Ambrose BK , Conway KP , Borek N , Taylor K , Goniewicz ML , Tobacco-product use by adults and youths in the United States in 2013 and 2014. N Engl J Med 2017;376(4):342–53. Erratum in: N Engl J Med 2018;378(5):492. 10.1056/NEJMsa1607538 28121512 PMC5317035

[6] Wang TW , Gentzke A , Sharapova S , Cullen KA , Ambrose BK , Jamal A . Tobacco product use among middle and high school students — United States, 2011–2017. MMWR Morb Mortal Wkly Rep 2018;67(22):629–33. 10.15585/mmwr.mm6722a3 29879097 PMC5991815

[7] Mays D , Hawkins KB , Bredfeldt C , Wolf H , Tercyak KP . The effects of framed messages for engaging adolescents with online smoking prevention interventions. Transl Behav Med 2017;7(2):196–203. 10.1007/s13142-017-0481-5 28290144 PMC5526817

[8] Centers for Disease Control and Prevention. Best practices for comprehensive tobacco control programs — 2014. Atlanta (GA): US Department of Health and Human Services, Centers for Disease Control and Prevention, National Center for Chronic Disease Prevention and Health Promotion, Office on Smoking and Health; 2014.

[9] Akl EA , Ward KD , Bteddini D , Khaliel R , Alexander AC , Lotfi T , The allure of the waterpipe: a narrative review of factors affecting the epidemic rise in waterpipe smoking among young persons globally. Tob Control 2015;24(Suppl 1):i13–21. 10.1136/tobaccocontrol-2014-051906 25618895 PMC4345979

[10] Arrazola RA , Singh T , Corey CG , Husten CG , Neff LJ , Apelberg BJ , Tobacco use among middle and high school students — United States, 2011–2014. MMWR Morb Mortal Wkly Rep 2015;64(14):381–5. 25879896 PMC5779546

[11] Pierce JP , Choi WS , Gilpin EA , Farkas AJ , Merritt RK . Validation of susceptibility as a predictor of which adolescents take up smoking in the United States. Health Psychol 1996;15(5):355–61. 10.1037/0278-6133.15.5.355 8891714

[12] Wellman RJ , Dugas EN , Dutczak H , O’Loughlin EK , Datta GD , Lauzon B , Predictors of the onset of cigarette smoking: a systematic review of longitudinal population-based studies in youth. Am J Prev Med 2016;51(5):767–78. 10.1016/j.amepre.2016.04.003 27180028

[13] Evans N , Farkas A , Gilpin E , Berry C , Pierce JP . Influence of tobacco marketing and exposure to smokers on adolescent susceptibility to smoking. J Natl Cancer Inst 1995;87(20):1538–45. 10.1093/jnci/87.20.1538 7563188

[14] Pierce JP , Choi WS , Gilpin EA , Farkas AJ , Berry CC . Tobacco industry promotion of cigarettes and adolescent smoking. JAMA 1998;279(7):511–5. Erratum in: JAMA 1998;280(5):422. 10.1001/jama.279.7.511 9480360

[15] Pierce JP , Distefan JM , Jackson C , White MM , Gilpin EA . Does tobacco marketing undermine the influence of recommended parenting in discouraging adolescents from smoking? Am J Prev Med 2002;23(2):73–81. 10.1016/S0749-3797(02)00459-2 12121794

[16] Bunnell RE , Agaku IT , Arrazola RA , Apelberg BJ , Caraballo RS , Corey CG , Intentions to smoke cigarettes among never-smoking US middle and high school electronic cigarette users: National Youth Tobacco Survey, 2011–2013. Nicotine Tob Res 2015;17(2):228–35. 10.1093/ntr/ntu166 25143298 PMC4515756

[17] Mays D , Gilman SE , Rende R , Luta G , Tercyak KP , Niaura RS . Influences of tobacco advertising exposure and conduct problems on smoking behaviors among adolescent males and females. Nicotine Tob Res 2014;16(6):855–63. 10.1093/ntr/ntu018 24590388 PMC4015102

[18] Mays D , Tercyak KP , Lipkus IM . The effects of brief waterpipe tobacco use harm and addiction education messages among young adult waterpipe tobacco users. Nicotine Tob Res 2016;18(5):777–84. 10.1093/ntr/ntv223 26438650 PMC5942619

[19] Primack BA , Sidani J , Agarwal AA , Shadel WG , Donny EC , Eissenberg TE . Prevalence of and associations with waterpipe tobacco smoking among U.S. university students. Ann Behav Med 2008;36(1):81–6. 10.1007/s12160-008-9047-6 18719977 PMC3004534

[20] Lipkus IM , Mays D , Tercyak KP . Characterizing young adults’ susceptibility to waterpipe tobacco use and their reactions to messages about product harms and addictiveness. Nicotine Tob Res 2017;19(10):1216–23. 10.1093/ntr/ntw251 27799355 PMC5896494

[21] Huang LL , Lazard AJ , Pepper JK , Noar SM , Ranney LM , Goldstein AO . Impact of The Real Cost campaign on adolescents’ recall, attitudes, and risk perceptions about tobacco use: a national study. Int J Environ Res Public Health 2017;14(1):42. 10.3390/ijerph14010042 28054993 PMC5295293

[22] Robinson J , Wang B , Jackson K , Donaldson E , Ryant C . Characteristics of hookah tobacco smoking sessions and correlates of use frequency among US adults: findings from wave 1 of the Population Assessment of Tobacco and Health (PATH) study. Nicotine Tob Res 2018;20(6):731–40. 10.1093/ntr/ntx060 28340148

[23] Demissie Z , Everett Jones S , Clayton HB , King BA . Adolescent risk behaviors and use of electronic vapor products and cigarettes. Pediatrics 2017;139(2):e20162921. 10.1542/peds.2016-2921 28115539 PMC10962496

[24] Food and Drug Administration, HHS. Deeming tobacco products to be subject to the Federal Food, Drug, and Cosmetic Act, as amended by the Family Smoking Prevention and Tobacco Control Act; restrictions on the sale and distribution of tobacco products and required warning statements for tobacco products. Final rule. Fed Regist 2016;81(90):28973–9106. 27192730

[25] Office on Smoking and Health, National Center for Chronic Disease Prevention and Health Promotion, Centers for Disease Control and Prevention. Hookahs. https://www.cdc.gov/tobacco/data_statistics/fact_sheets/tobacco_industry/hookahs/index.htm. Updated 2016. Accessed July 17, 2018.

[26] Office on Smoking and Health, National Center for Chronic Disease Prevention and Health Promotion, Centers for Disease Control and Prevention. Youth and tobacco use. https://www.cdc.gov/tobacco/data_statistics/fact_sheets/youth_data/tobacco_use/index.htm. Updated 2018. Accessed July 17, 2018.

[27] Evans WD , Rath JM , Hair EC , Snider JW , Pitzer L , Greenberg M , Effects of the *truth FinishIt* brand on tobacco outcomes. Prev Med Rep 2017;9:6–11. 10.1016/j.pmedr.2017.11.008 29276667 PMC5724797

[28] Krauss MJ , Sowles SJ , Moreno M , Zewdie K , Grucza RA , Bierut LJ , Hookah-related Twitter chatter: a content analysis. Prev Chronic Dis 2015;12:E121. 10.5888/pcd12.150140 26226068 PMC4523113

[29] Primack BA , Carroll MV , Shensa A , Davis W , Levine MD . Sex differences in hookah-related images posted on Tumblr: a content analysis. J Health Commun 2016;21(3):366–75. 10.1080/10810730.2015.1095814 26890733 PMC4873310

